# High Body Mass Index and Triglycerides Help Protect against Osteoporosis in Patients with Type 2 Diabetes Mellitus

**DOI:** 10.1155/2020/1517879

**Published:** 2020-10-27

**Authors:** Hang Zhao, Chong Zheng, Kexin Gan, Cuijuan Qi, Luping Ren, Guangyao Song

**Affiliations:** ^1^Endocrinology Department, Hebei General Hospital, 348, Heping West Road, Shijiazhuang, Hebei 050051, China; ^2^Pediatric Orthopaedics, The Third Hospital of Shijiazhuang, 15, Sports South Street, Shijiazhuang, Hebei 050011, China

## Abstract

**Purpose:**

This study was conducted to investigate whether high body mass index (BMI) and triglycerides (TGs) were protective factors for reducing osteoporosis (OP) in patients with type 2 diabetes mellitus (T2DM). *Participants and Methods*. Seventy-nine patients (aged 20 to 81) with T2DM were included in the study. Basic information and blood indicators were collected. Bone mineral density was used to diagnose OP. Participants were grouped according to BMI (normal weight vs. overweight/obese participants), TG (normal TG vs. hypertriglyceridemia), and OP (non-OP vs. OP), and differences were compared between groups. Regression analysis was used to explore whether BMI or TG were independent factors affecting OP.

**Results:**

The proportions of OP in the overweight/obese and hypertriglyceridemic groups were significantly lower than those in the normal weight (30.0% vs. 69.0%; *P* = 0.001) and normal TG (27.3% vs. 56.5%; *P* = 0.010) groups. In the OP group, the BMI (24.8 ± 3.4 vs. 26.6 ± 2.5 kg/m^2^; *P* = 0.009) was significantly lower than that in the non-OP group, and TG showed the same trend (1.30 (0.81) vs. 1.71 (1.1) mmol/L; *P* = 0.020). Logistic regression in the crude model showed that the odds ratios (ORs) of OP in the overweight/obese and hypertriglyceridemic groups were 0.193 (95% CI: 0.071, 0.520) and 0.315 (95% CI: 0.119, 0.830) compared with those of the normal weight and normal TG groups. After adjusting for sex and smoking, the ORs were 0.204 (95% CI: 0.074, 0.567) and 0.242 (95% CI: 0.082, 0.709) for the overweight/obese and hypertriglyceridemic groups, respectively. After adjusting for all confounding factors, the ORs for these groups were 0.248 (95% CI: 0.083, 0.746) and 0.299 (95% CI: 0.091, 0.989), respectively.

**Conclusion:**

BMI and TG are independent protective factors against OP in patients with T2DM.

## 1. Introduction

Type 2 diabetes mellitus (T2DM) is a global disease, and its complications including cardiovascular, cerebrovascular, kidney, and eyes have resulted in heavy health and economic burdens on patients, families, and society [1]. Osteoporosis (OP) and diabetes (DM) are metabolic diseases that often occur in the elderly. OP is also a skeletal complication of T2DM [2]. OP is a systemic bone disease characterized by reduced bone mass and damaged bone tissue microstructure, leading to increased bone fragility and being prone to fracture [3]. Osteoporotic fractures cause great harm and are a main cause of disability and death in elderly patients [4]. Within 1 year after hip fracture, 20% of patients will die from complications such as limb venous thrombosis and fallopian pneumonia, approximately 50% will become disabled, and their quality of life will be reduced [5]. Medical treatment and nursing for OP and fractures require substantial manpower, material, and financial resources.

A cross-sectional study including 200 subjects in India found that the prevalence of OP in patients with T2DM is about 20-35% [6]. The risk of hip fracture is 2.5-fold higher than general population in patients with T2DM [7]. Thus, OP in patients with T2DM must be prevented as early as possible.

Some previous studies have examined the relationship between BMI and blood lipids as well as bone mineral density (BMD) and OP in patients with non-T2DM. For example, a study by Wu et al. found that with the increasing of BMI, the risk of OP was reduced in the Chinese adult population [8]. Compared with high-density lipoprotein cholesterol (HDL − C) ≤ 1.55 mmol/L, subjects with HDL-C levels of ≥1.56 mmol/L had a greater prevalence of OP [9]. But the studies there are few studies about the relationship between BMI/lipid and OP in patients with T2DM. Thus, our study was conducted to investigate whether high BMI and blood lipid levels are protective factors against OP in patients with T2DM.

## 2. Participants and Methods

### 2.1. Participants and Study Design

**Figure 1:**
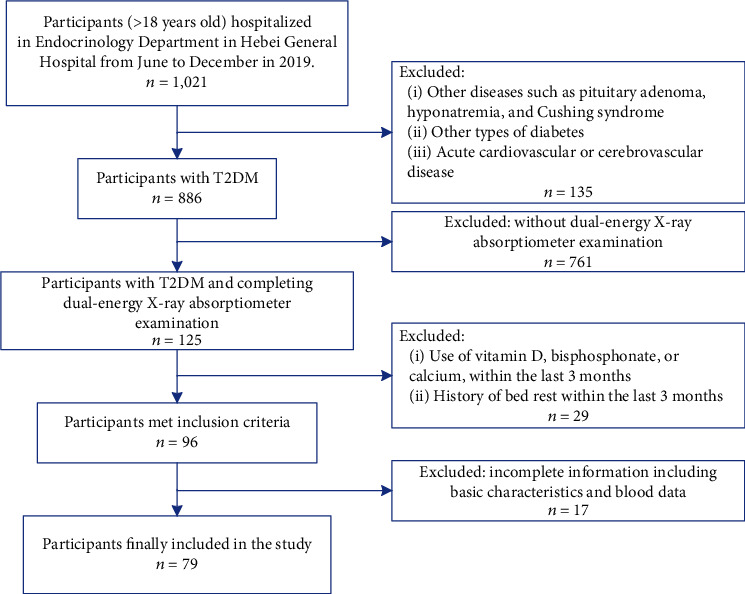
The flow chart of the study.

This was a case-control study. Participants (aged > 18 years old) were recruited between June 2019 and December 2019. After screening gradually, the study included 79 participants (aged 10 to 81 years old) including 44 males and 35 females who were hospitalized in Hebei General Hospital (see flow chart: Figure 1) (Clinical Trial Registry Number: ChiCTR2000029391). The Hebei General Hospital Ethics Committee approved the study.

Inclusion criteria were (1) clinically diagnosed T2DM meeting the diagnostic criteria proposed by the American Diabetes Association in 2014 [10], (2) BMI ≥ 18.5 kg/m^2^, and (3) BMD tests were performed, and the OP standards were in line with those of the World Health Organization (based on dual-energy X-ray absorptiometer: normal: –1 ≤ T-score, osteopenia: –2.5 < T-score < –1, and OP: ≤–2.5 T-score) [11].

Exclusion criteria were (1) other types of DM such as type 1 diabetes or monogenic diabetes, (2) patients who had a fall or fracture within the last 3 months, (3) use of vitamin D, calcium, or bisphosphonate within the last 3 months, (4) history of bed rest within the last 3 months, and (5) acute cardiovascular or cerebrovascular disease within the last 3 months.

### 2.2. Data Collection

One author collected the patients' basic information, including sex, age, duration of T2DM, family history of DM (relatives within three generations), smoking history (continuous or cumulative regular smoking for at least 6 months or older, with an average of more than one cigarette per day), drinking history (continuous or cumulative consuming alcohol for at least 6 months, with more than 50 g per day converted to white wine), hypertension history (systolic blood pressure ≥ 140 mmHg or/and diastolic blood pressure ≥ 90 mmHg), gastrointestinal disease, and BMI (body weight (kg)/height (m^2^)). The participants were fasted overnight for more than 8–10 hours before blood was collected the following morning from a cephalic vein. Blood indicators included the lipid profile (total cholesterol (TC), triglycerides (TGs), high-density lipoprotein cholesterol (HDL-C), low-density lipoprotein cholesterol (LDL-C), very-low-density lipoprotein cholesterol (VLDL-C), apolipoprotein A1 (ApoA1), and apolipoprotein B (ApoB)), HbA1c, total protein, albumin, urea nitrogen, creatinine (Cr), 25-hydroxyvitamin D (25OHD), OC (osteocalcin), *β*-C-terminal cross-linked telopeptide of type I collagen (*β*-CTX), P1NP, and parathyroid hormone (PTH). Blood tests including lipids were performed in the same laboratory using the Hitachi 7600 Automatic Analyzer (HITACHI, Japan).

### 2.3. Participant Groupings

Our study included three groupings. First, BMI was calculated from the height and body weight as [BMI (kg/m^2^) = weight (kg)/height (m^2^)]. The subjects were then divided into two groups according to BMI: the normal weight group (18.5–24.9 kg/m^2^) and the overweight/obese group (25.0–29.9 kg/m^2^). Second, the subjects were divided into the normal TG group (≤1.7 mmol/L) and the hypertriglyceridemic group (>1.7 mmol/L). Third, the subjects were grouped into the nonosteoporosis and osteoporosis groups.

### 2.4. Statistical Analysis

Differences between groups were tested via variance analysis/nonparametric tests for continuous variables or with a chi-square test for proportional variables and are expressed as the mean ± standard deviation and median (interquartile range), respectively. Logistic regression was used to analyze the associations between BMI/TG and OP. Three models were fitted for the outcomes. In model 1, a crude odds ratio (OR) and 95% confidence interval (95% CI) were obtained by adding the independent variable into the equation. In model 2, potential confounding variables, including age and sex, were adjusted. Model 3 was further adjusted by including hypertension, BMI/TG, and Cr. Statistical significance was accepted at *P* < 0.05. All statistical analyses were performed in SPSS 22.0 (IBM, Inc., Armonk, NY, USA).

## 3. Results

### 3.1. Clinical Characteristics of the Participants according to BMI Category

**Figure 2:**
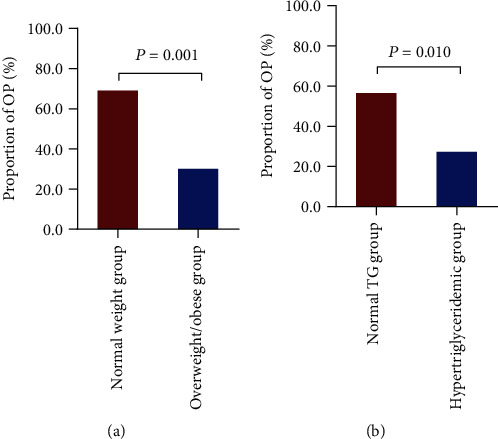
Proportion of OP in different groups. Note: (a) proportion of OP in the normal weight and overweight/obese groups and (b) proportion of OP in the normal TG and hypertriglyceridemic groups. Abbreviations: OP: osteoporosis; TG: triglyceride.

The proportion of OP in the overweight/obese group in patients with T2DM was 30%, which was significantly lower than that of the normal weight group (69%) (*P* = 0.001) (Figure 2(a)). In addition, compared with the normal weight group, age and HDL-C were significantly decreased in the overweight/obese group (*P* = 0.019 and 0.031, respectively). Bone turnover markers (e.g., 25OHD and OC) did not differ between groups (Table 1).

### 3.2. Clinical Characteristics of Participants according to TG Category

The hypertriglyceridemic group had a significantly lower OP incidence (27.3%) than did the normal TG group (56.5%; *P* = 0.010) (Figure 2(b)). Compared with the normal TG group, the incidence of hypertension in the hypertriglyceridemic group was significantly increased (60.6% vs. 34.8%, *P* = 0.023), and BMI, TC, LDL-C, VLDL-C, and ApoB showed the same trend. The bone turnover markers, OC, *β*-CTX, and P1NP, were significantly lower in the hypertriglyceridemic group than in the normal TG group (Table 2).

### 3.3. Clinical Characteristics of Participants according to OP Category

**Figure 3:**
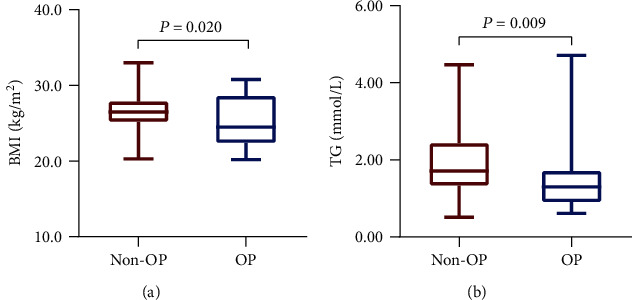
Comparisons of BMI and TG between the non-OP and OP groups. Note: (a) BMI level in the non-OP and OP groups and (b) TG level in the non-OP and OP groups. Abbreviations: BMI: body mass index; OP: osteoporosis; TG: triglyceride.

Participants were grouped according to whether they had OP. The proportion of women in the OP group (57.1%) was significantly higher than that in the non-OP group (34.1%; *P* = 0.040). However, the OP group had lower percentages of hypertension and smoking histories. The BMI (24.8 ± 3.4 vs. 26.6 ± 2.5 kg/m^2^) and TG (1.30 (0.81) vs. 1.71 (1.1) mmol/L) in the OP group were significantly lower than those in the non-OP group (*P* = 0.009, *P* = 0.020) (Figures 3(a) and 3(b)), and Cr (64.98 ± 11.08 vs. 72.13 ± 13.26 *μ*mmol/L) showed the same trend (*P* = 0.014). Bone turnover markers did not statistically differ between the groups (Table 3).

### 3.4. Associations between BMI and Risk of OP

Comparing the differences between the OP and non-OP groups showed that sex, hypertension, smoking, BMI, TG, and Cr may affect OP. We used logistic regression to explore whether BMI is an independent factor affecting OP. Unadjusted confounding factors revealed that the risk of OP in the overweight/obese group was 0.193 times than that of the normal weight group (95% CI: 0.071–0.520, *P* = 0.001). After adjusting for sex and smoking, the risk was 0.204 times greater (95% CI: 0.074–0.567, *P* = 0.002). After adjusting for all confounding factors, the risk was 0.248 times (95% CI: 0.083–0.746, *P* = 0.013) (Table 4).

### 3.5. Associations between TG and Risk of OP

In the crude model, the OR of OP in the hypertriglyceridemic group was 0.315 (95% CI: 0.119–0.830, *P* = 0.020) compared with that of the normal TG group. In model 2, sex and smoking were adjusted, and the OR was 0.242 (95% CI: 0.082–0.709, *P* = 0.010). In model 3, based on model 2, hypertension, BMI, and Cr were adjusted, and the OR was 0.299 (95% CI: 0.091–0.989, *P* = 0.048) (Table 5).

## 4. Discussion

Malnutrition can cause or exacerbate many diseases such as OP. BMI is an important indicator for assessing patients' nutritional status [12, 13]. Some studies have shown that BMI and BMD are positively correlated [14, 15]. Low weight and large sudden weight loss can lead to bone loss [16]. An American study suggested that increased BMI can prevent a decrease in BMD, especially for patients with OP, where a 10-unit increase in BMI will help BMD levels return to normal [17]. Saarelainen et al. found that women with a BMI of 20 kg/m^2^ had reduced bone mass at the spine and femoral neck at 2 and 4 years after menopause, whereas women with a BMI of 30 kg/m^2^ had reduced bone mass at the spine and femoral neck at 5 and 9 years after menopause [18]. Some studies have suggested that dyslipidemia may lead to increased bone resorption and reduced bone formation [19]. In this study, we chose triglycerides as a potential variable because blood lipid levels can indicate nutritional status especially triglycerides are closely related to diet. In addition, the association of triglycerides and osteoporosis was controversial in previous studies. A study involving 214 postmenopausal women in Japan found that lower TG was associated with an increased risk of vertebral fractures [20]. Dennison et al. observed a significant positive correlation between fasting TG levels and lumbar spine BMD [21]. However, a Korean study found no association between serum lipid profiles and BMD in postmenopausal women [22]. In our study, triglycerides differed between the nonosteoporosis and osteoporosis groups. Thus, we added triglycerides to the regression analysis.

BMI and TG may be protective factors of OP in this study. High BMI and obesity can affect bone density and osteoporosis via several mechanisms. First, the bones of people with a higher BMI are under greater gravity stress. Second, adipocytes and osteoblasts have the same tendency to differentiate into adipocytes or osteoblasts under the action of transcription factors because they originate from pluripotent mesenchymal stem cells [23]. Third, adipose tissue secretes various cytokines, such as resistin, leptin, and adiponectin [24], which may send signals through the central nervous system and regulate bone turnover through autonomic innervation [25]. Obesity and blood lipids are closely related. People with high BMI often have metabolic syndromes such as insulin resistance and hyperlipidemia. Therefore, the mechanism by which high blood lipids affect bone metabolism is related to BMI, especially fat cells. Additionally, TG may form a layer between collagen fibers and mineral crystals and can mediate protein matrix attachment, thereby increasing bone stability [26].

Obesity is caused by excessive fat accumulation in the body and is the main cause of comorbidities such as T2DM and cardiovascular disease, which increase morbidity and mortality [27]. High BMI and hyperlipidemia are risk factors or progression factors for DM [28, 29]. Weight loss and lipid control remain the main strategies for preventing or treating T2DM and cardiovascular/cerebrovascular diseases and reducing their related risks [30]. However, this study was conducted to investigate whether BMI and TG are protective factors against OP in patients with T2DM. After grouping normal weight vs. overweight/obese patients and those with normal TG vs. hypertriglyceridemia, both BMI and TG were protective factors against OP in patients with T2DM, regardless of corrections for confounding factors. Consistent with these results, the OP incidence was significantly increased in the overweight/obese and hypertriglyceridemic groups compared with that in the normal group. High BMI and TG may represent good nutritional status. However, evidence-based medicine has shown that being overweight or hyperlipidemic can worsen the complications of T2DM and cardiovascular/cerebrovascular diseases. Methods of reducing the risk of OP or fracture while also reducing or delaying the progression of T2DM complications require further study. The appropriate range for BMI and TG for reducing both OP and T2DM risk should be determined.

This study has some limitations. First, the sample size was small. A larger sample should be used in future studies to obtain more robust results. Second, we grouped BMI into the normal and overweight/obese groups but did not include thinner participants. In our next work, we should categorize BMI into more detailed groups: thinner, normal, overweight, and obese. Third, our study only included participants with non-OP and OP, but did not include participants with low BMD (osteopenia); it is important to investigate the relationship between different BMD status (normal BMD, osteopenia, and OP) and BMI or TG. Fourth, men and women were included in this study but OP incidence in menopausal women may differ from that in men and nonmenopausal women owing to changes in hormone levels. It is appropriate to perform subgroup analysis (men, menopausal women, and nonmenopausal women). Fifth, homocysteine, a metabolic indicator, is usually included in studies involved in DM and cardiovascular and cerebrovascular diseases. However, it was not included in this study. Last, in future studies, we will investigate both genetic and environmental factors, because they are thought to affect the association of BMI and blood lipids with osteoporosis.

In summary, our study showed that high BMI and TG may reduce the risk of OP in patients with T2DM, and higher BMI and TG may be protective factors.

## Figures and Tables

**Table 1:** Clinical characteristics of patients with T2DM by BMI category.

	Normal weight group (20–24.9 kg/m^2^)	Overweight/obese group (≥25 kg/m^2^)	*P* value
*n*	29	50	
Sex (F, %)	25 (51.7%)	20 (40%)	0.312
Age (years)	59.0 ± 8.6	53.6 ± 10.4	0.019
DM course (years)			
0-1	7 (24.1%)	13 (26.0%)	
1-10	14 (48.3%)	21 (42.0%)	0.858
>10	8 (27.6%)	16 (32.0%)	
History of DM	10 (34.5%)	16 (32.0%)	0.821
Hypertension	11 (37.9%)	25 (50.0%)	0.299
Gastrointestinal disorders	4 (13.8%)	6 (12.0%)	1.000
Smoking	7 (24.1%)	19 (38.0%)	0.206
Drinking	6 (20.7%)	19 (38.0%)	0.111
HbA1c (%)	9.6 (4.2)	9.0 (2.4)	0.376
TC (mmol/L)	4.84 ± 1.17	4.55 ± 1.04	0.268
TG (mmol/L)	1.41 (1.01)	1.59 (1.18)	0.095
HDL-C (mmol/L)	1.13 ± 0.21	1.02 ± 0.21	0.031
LDL-C (mmol/L)	3.18 ± 0.74	3.00 ± 0.75	0.303
VLDL-C (mmol/L)	0.46 (0.22)	0.47 (0.38)	0.508
ApoA1 (mmol/L)	1.32 ± 0.21	1.27 ± 0.19	0.294
ApoB (mmol/L)	0.82 ± 0.25	0.81 ± 0.19	0.831
Total protein (g/L)	68.53 ± 6.46	68.06 ± 5.90	0.743
Albumin (g/L)	40.95 ± 2.86	41.41 ± 2.29	0.749
BUN (mmol/L)	4.91 ± 1.27	5.14 ± 1.30	0.442
Cr (*μ*mmol/L)	66.35 ± 12.02	70.44 ± 13.07	0.180
25OHD (ng/mL)	13.18 (9.81)	15.25 (9.92)	0.248
OC (ng/mL)	15.05 ± 4.09	14.18 ± 5.26	0.421
*β*-CTX (ng/mL)	0.40 (0.29)	0.38 (0.33)	0.827
P1NP (ng/mL)	38.77 (17.46)	42.76 (29.99)	0.935
PTH (pg/mL)	41.55 ± 14.00	41.19 ± 14.89	0.915
OP	20 (69.0%)	15 (30.0%)	0.001

Note: data were expressed as number (%) or mean ± SD/median (interquartile range). Abbreviations: ApoA1: apolipoprotein A1; ApoB: apolipoprotein B; BMD: bone mineral density; BMI: body mass index; BUN: blood urea nitrogen; Cr: creatinine; DM: diabetes mellitus; HbA1c: glycated hemoglobin; HDL-C: high-density lipoprotein cholesterol; LDL-C: low-density lipoprotein cholesterol; T2DM: type 2 diabetes mellitus; TC: total cholesterol; TG: triglyceride; VLDL-C: very-low-density lipoprotein cholesterol; OC: osteocalcin; OP: osteoporosis; P1NP: procollagen type 1 N-terminal propeptide; PTH: parathyroid hormone; 25OHD: 25-hydroxyvitamin D; *β*-CTX: *β*-C-terminal cross-linked telopeptide of type I collagen.

**Table 2:** Clinical characteristics of patients with T2DM by TG category.

	Normal TG group (≤1.7 mmol/L)	Hypertriglyceridemic group (>1.7 mmol/L)	*P* value
*n*	46	33	
Gender (F, %)	18 (39.1%)	17 (51.5%)	0.274
Age (years)	55.7 ± 10.2	55.4 ± 10.0	0.914
DM course (years)			
0-1	14 (30.4%)	6 (18.2%)	
1-10	19 (41.3%)	16 (48.5%)	0.466
>10	13 (28.3%)	11 (33.3)	
History of DM	14 (30.4%)	12 (36.4%)	0.580
Hypertension	16 (34.8%)	20 (60.6%)	0.023
Gastrointestinal disorders	5 (10.9%)	5 (15.2%)	0.734
Smoking	14 (30.4%)	12 (36.4%)	0.580
Drinking	14 (30.4%)	11 (33.3%)	0.785
BMI (kg/m^2^)	25.2 ± 2.9	26.8 ± 2.7	0.017
HbA1c (%)	9.0 ± 2.4	9.2 ± 1.9	0.801
TC (mmol/L)	4.40 ± 0.95	5.01 ± 1.18	0.014
HDL-C (mmol/L)	1.08 ± 0.22	1.03 ± 022	0.321
LDL-C (mmol/L)	2.89 ± 0.72	3.31 ± 0.73	0.015
VLDL-C (mmol/L)	0.39 (0.24)	0.65 (0.43)	0.000
ApoA1 (mmol/L)	1.26 ± 0.18	1.33 ± 0.21	0.158
ApoB (mmol/L)	0.75 ± 0.17	0.90 ± 0.23	0.002
Total protein (g/L)	67.13 ± 5.74	69.78 ± 6.28	0.056
Albumin (g/L)	41.28 ± 2.61	41.19 ± 2.40	0.877
BUN (mmol/L)	5.22 ± 1.29	4.81 ± 1.25	0.158
Cr (*μ*mmol/L)	68.42 ± 11.97	69.65 ± 13.95	0.682
25OHD (ng/mL)	16.73 (9.72)	13.61 (7.01)	0.120
OC (ng/mL)	15.55 ± 4.85	12.97 ± 4.54	0.020
*β*-CTX (ng/mL)	0.49 (0.32)	0.32 (0.24)	0.007
P1NP (ng/mL)	47.32 (25.95)	34.79 (20.77)	0.007
PTH (pg/mL)	40.81 ± 13.22	42.03 ± 16.15	0.714
OP	26 (56.5%)	9 (27.3%)	0.010

Note: data were expressed as number (%) or mean ± SD/median (interquartile range). Abbreviations: ApoA1: apolipoprotein A1; ApoB: apolipoprotein B; BMD: bone mineral density; BMI: body mass index; BUN: blood urea nitrogen; Cr: creatinine; DM: diabetes mellitus; HbA1c: glycated hemoglobin; HDL-C: high-density lipoprotein cholesterol; LDL-C: low-density lipoprotein cholesterol; T2DM: type 2 diabetes mellitus; TC: total cholesterol; TG: triglyceride; VLDL-C: very-low-density lipoprotein cholesterol; OC: osteocalcin; OP: osteoporosis; P1NP: procollagen type 1 N-terminal propeptide; PTH: parathyroid hormone; 25OHD: 25-hydroxyvitamin D; *β*-CTX: *β*-C-terminal cross-linked telopeptide of type I collagen.

**Table 3:** Clinical characteristics of patients with T2DM by OP category.

	Non-OP group	OP group	*P* value
*n*	44	35	
Gender (F, %)	15 (34.1%)	20 (57.1%)	0.040
Age (years)	54.2 ± 8.4	57.3 ± 11.7	0.186
BMI (kg/m^2^)	26.6 ± 2.5	24.8 ± 3.4	0.009
Duration of DM (years)			
0-1	12 (15.2%)	8 (10.1%)	
1-10	17 (21.5)	18 (22.8%)	0.517
>10	15 (19.0%)	9 (11.4%)	
History of DM	12 (15.2%)	14 (17.7%)	0.232
Hypertension	25 (56.8%)	11 (31.4%)	0.024
Gastrointestinal disorders	6 (7.6%)	4 (5.1%)	1.000
Smoking	19 (43.2)	7 (8.9%)	0.029
Drinking	17 (21.5%)	8 (10.1%)	0.134
HbA1c (%)	9.2 ± 2.0	8.9 ± 2.4	0.564
TC (mmol/L)	4.57 ± 1.17	4.77 ± 0.99	0.407
TG (mmol/L)	1.71 (1.1)	1.30 (0.81)	0.020
HDL-C (mmol/L)	1.02 ± 0.23	1.11 ± 0.19	0.078
LDL-C (mmol/L)	3.01 ± 0.86	3.13 ± 0.75	0.535
VLDL-C (mmol/L)	0.53 (0.39)	0.52 (0.29)	0.678
ApoA1 (mmol/L)	1.30 ± 0.20	1.29 ± 0.20	0.836
ApoB (mmol/L)	0.82 ± 0.21	0.81 ± 0.21	0.873
Total protein (g/L)	68.33 ± 6.15	68.12 ± 6.08	0.877
Albumin (g/L)	41.41 ± 2.41	41.03 ± 2.65	0.515
BUN (mmol/L)	5.27 ± 1.42	4.79 ± 1.06	0.095
Cr (*μ*mmol/L)	72.13 ± 13.26	64.98 ± 11.08	0.014
25OHD (ng/mL)	15.18 (10.66)	15.04 (9.41)	0.582
OC (ng/mL)	14.07 ± 5.13	15.03 ± 4.51	0.389
*β*-CTX (ng/mL)	0.37 (0.33)	0.42 (0.29)	0.460
P1NP (ng/mL)	38.10 (27.61)	47.32 (22.74)	0.106
PTH (pg/mL)	43.74 ± 14.74	38.27 ± 13.62	0.095

Note: data were expressed as number (%) or mean ± SD/median (interquartile range). Abbreviations: ApoA1: apolipoprotein A1; ApoB: apolipoprotein B; BMD: bone mineral density; BMI: body mass index; BUN: blood urea nitrogen; Cr: creatinine; DM: diabetes mellitus; HbA1c: glycated hemoglobin; HDL-C: high-density lipoprotein cholesterol; LDL-C: low-density lipoprotein cholesterol; T2DM: type 2 diabetes mellitus; TC: total cholesterol; TG: triglyceride; VLDL-C: very-low-density lipoprotein cholesterol; OC: osteocalcin; OP: osteoporosis; P1NP: procollagen type 1 N-terminal propeptide; PTH: parathyroid hormone; 25OHD: 25-hydroxyvitamin D; *β*-CTX: *β*-C-terminal cross-linked telopeptide of type I collagen.

**Table 4:** Logistic regression model of BMI categories for osteoporosis in patients with T2DM adjusting for covariates.

	Normal weight group	Overweight/obese group	*P* for trend
No. of OP subjects	29	50	
Model 1	1.00 (reference)	0.193 (0.071, 0.520)	0.001
Model 2	1.00 (reference)	0.204 (0.074, 0.567)	0.002
Model 3	1.00 (reference)	0.248 (0.083, 0.746)	0.013

Note: data were expressed as OR (95CI). Model 1: crude model; model 2: adjusted for gender and smoking; model 3: adjusted for gender, smoking, hypertension, TG, and Cr. Abbreviations: Cr: creatinine; OR: odds ratio; T2DM: type 2 diabetes mellitus; TG: triglyceride; 95CI: 95% confidence interval.

**Table 5:** Logistic regression model of TG categories for osteoporosis in patients with T2DM adjusting for covariates.

	Normal TG group	Hypertriglyceridemic group	*P* for trend
No. of subjects	46	33	
Model 1	1.00 (reference)	0.315 (0.119, 0.830)	0.020
Model 2	1.00 (reference)	0.242 (0.082, 0.709)	0.010
Model 3	1.00 (reference)	0.299 (0.091, 0.989)	0.048

Note: data were expressed as OR (95CI). Model 1: crude model; model 2: adjusted for gender and smoking; model 3: adjusted for gender, smoking, hypertension, TG, and Cr. Abbreviations: Cr: creatinine; OR: odds ratio; T2DM: type 2 diabetes mellitus; TG: triglyceride; 95CI: 95% confidence interval.

## Data Availability

The original data can be requested by email.

## Abbreviations

ApoA1: Apolipoprotein A1
ApoB: Apolipoprotein B
BMD: Bone mineral density
BMI: Body mass index
BUN: Blood urea nitrogen
Cr: Creatinine
DM: Diabetes mellitus
HbA1c: Glycated hemoglobin
HDL-C: High-density lipoprotein cholesterol
LDL-C: Low-density lipoprotein cholesterol
T2DM: Type 2 diabetes mellitus
TC: Total cholesterol
TG: Triglyceride
VLDL-C: Very-low-density lipoprotein cholesterol
OC: Osteocalcin
OP: Osteoporosis
OR: Odds ratio
P1NP: Procollagen type 1 N-terminal propeptide
PTH: Parathyroid hormone
25OHD: 25-Hydroxyvitamin D
95% CI: 95% confidence interval
*β*-CTX: *β*-C-Terminal cross-linked telopeptide of type I collagen.
